# Delayed Presentation of Drug-Induced Hepatic Injury

**DOI:** 10.7759/cureus.9713

**Published:** 2020-08-13

**Authors:** Shohana Ahmed, Nirmal K Onteddu, Ali Jabur, Sai Swarupa R Vulasala, Swapna Kolli

**Affiliations:** 1 Internal Medicine, Texas Tech University Health Sciences Center at Permian Basin, Odessa, USA; 2 Internal Medicine/Hospitalist, Flowers Hospital, Dothan, USA; 3 Medicine, Sri Venkateswara Medical College, Tirupati, IND; 4 Internal Medicine, Texas Tech University Health Sciences Center at the Permian Basin, Odessa, USA

**Keywords:** drug-induced liver disease, amoxicillin – clavulanate, udca therapy

## Abstract

Drug-induced hepatotoxicity is a major cause of acute liver failure (ALF) in the United States. There are many offending agents like prescription drugs and herbal remedies. However, the most common prescription medication involved worldwide is amoxicillin-clavulanate. We report an unusually delayed presentation of severe cholestatic hepatitis caused by amoxicillin-clavulanate in a 20-year-old female with worsening hyperbilirubinemia that was successfully treated with corticosteroids and ursodeoxycholic acid (UDCA).

## Introduction

With an incidence of 10 cases per 100,000 persons, drug-induced hepatotoxicity is a major cause of acute liver failure (ALF) in the United States [1-3]. There are many offending agents like prescription drugs and herbal remedies [4]. However, the most common prescription medication involved worldwide is amoxicillin-clavulanate [2, 5]. The liver injury associated with amoxicillin-clavulanate usually manifests within an average of three to four weeks from initial ingestion [5-6]. While the exact pathophysiology is still unknown, the presence of eosinophilic infiltrates indicates that the cholestatic-type injury caused by the antibiotic may be attributed to immunological factors [5, 7]. Withdrawal of the offending agent is usually the optimal management, however, corticosteroid therapy has been used in cases with severe cholestatic injury [5]. The common risk factors include advanced age, male sex, ethanol intake, medical illness, as well as combination of other hepatotoxic drugs [4, 8-11].

## Case presentation

A 20-year-old female patient with no significant past medical history presented to our hospital with a five-day history of nausea, vomiting, epigastric pain, and yellowing of the skin. She denied exposure to any recent medications, herbal supplements, illicit drugs, and alcohol. Vitals were within normal limits except for sinus tachycardia. Pertinent physical exam findings were conjunctival icterus, epigastric tenderness, with no associated splenomegaly or hepatomegaly. Significant laboratory workup revealed leucocytes of 13.5 x 103/mcl, hemoglobin 13.8 g/dL, platelet count 250 x 103/mcl, prothrombin time 13.6 s, INR 1.04, albumin 3.6 g/dL, serum glutamic-oxaloacetic transaminase (SGOT) 39 U/L, serum glutamic pyruvic transaminase (SGPT) 57 U/L, alkaline phosphatase 207 U/L, total bilirubin 6.9 mg/dL, lipase 50 U/L, amylase 93 U/L, gamma glutamyl transpeptidase 141 U/L, ferritin 232 ng/mL, and ceruloplasmin 51 mg/dL. Serologies for acute hepatotropic viruses, Herpes simplex virus, Epstein-Barr virus, autoimmune markers such as antinuclear antibody (ANA), antimitochondrial antibodies (AMA), antineutrophil cytoplasmic antibody (ANCA) were negative. All other laboratory results including hematological, biochemical, and metabolic panels were within normal limits. 

**Figure 1:**
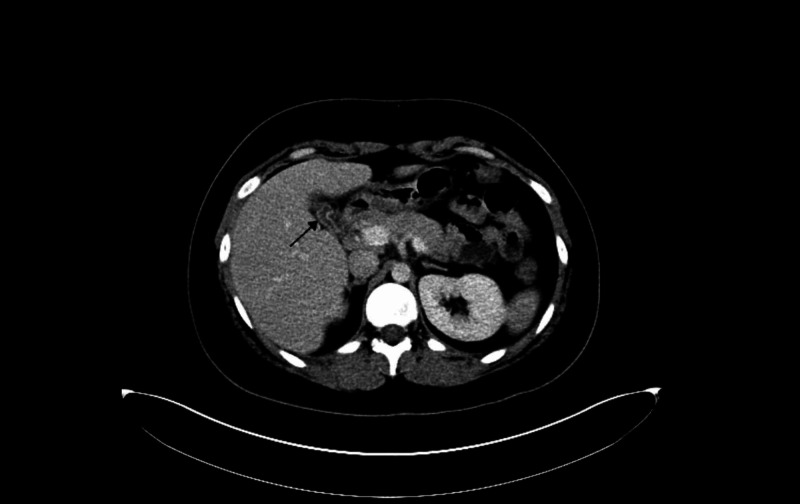
CT abdomen and pelvis showing contracted gall bladder.

**Figure 2:**
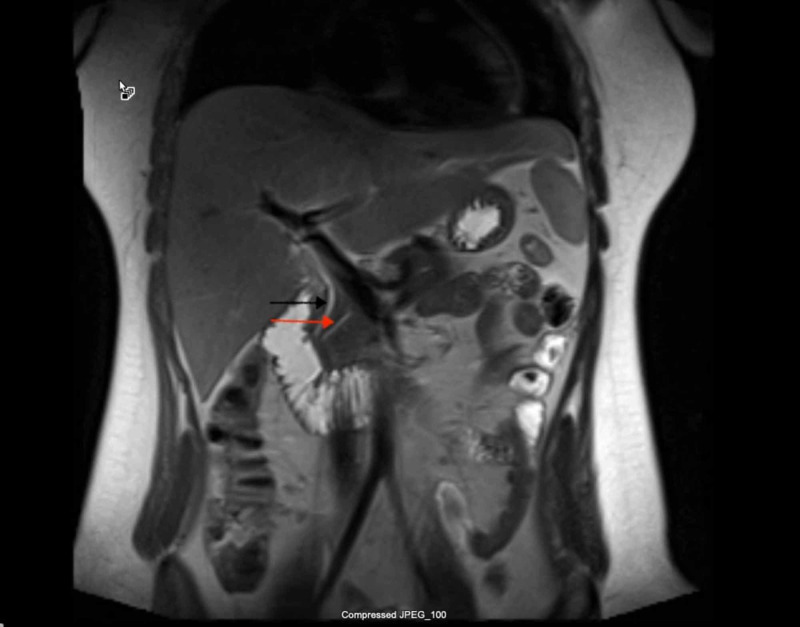
MRCP showing patent common bile duct (black arrow) and pancreatic duct (red arrow). MRCP, magnetic resonance cholangiopancreatography

**Figure 3:**
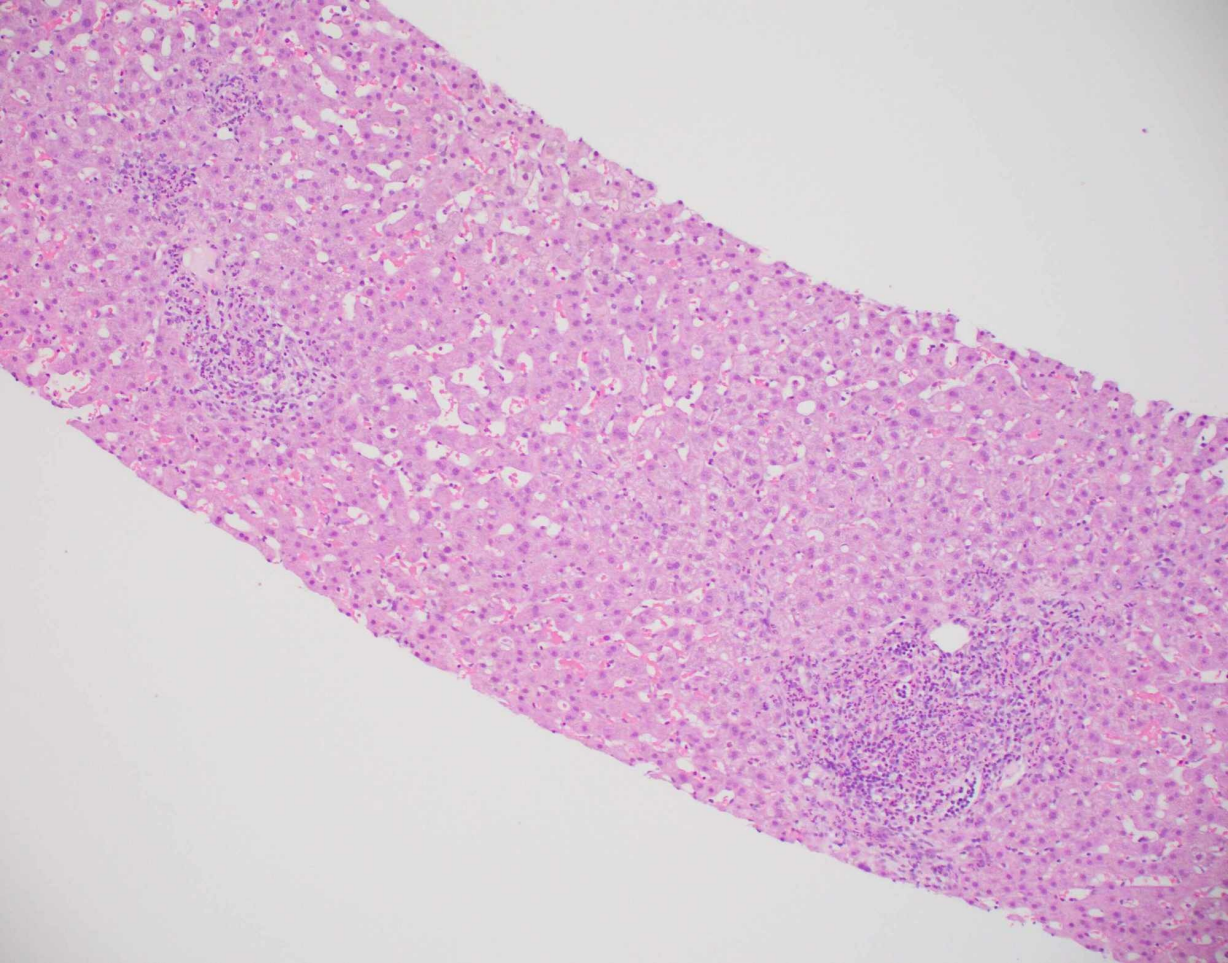
Inflammation of Zone 1 and Zone 3.

**Figure 4:**
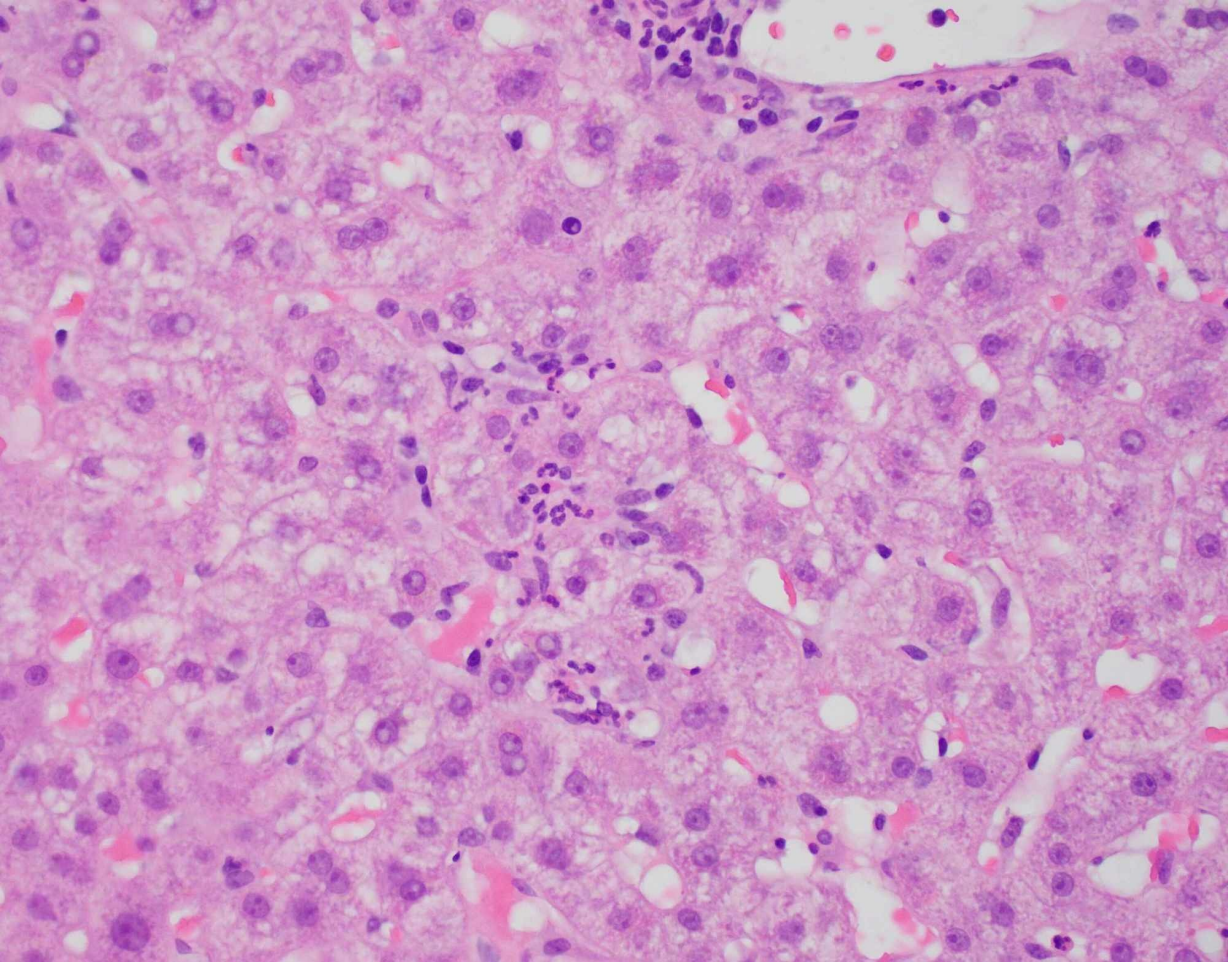
Acute lobular inflammation.

**Figure 5:**
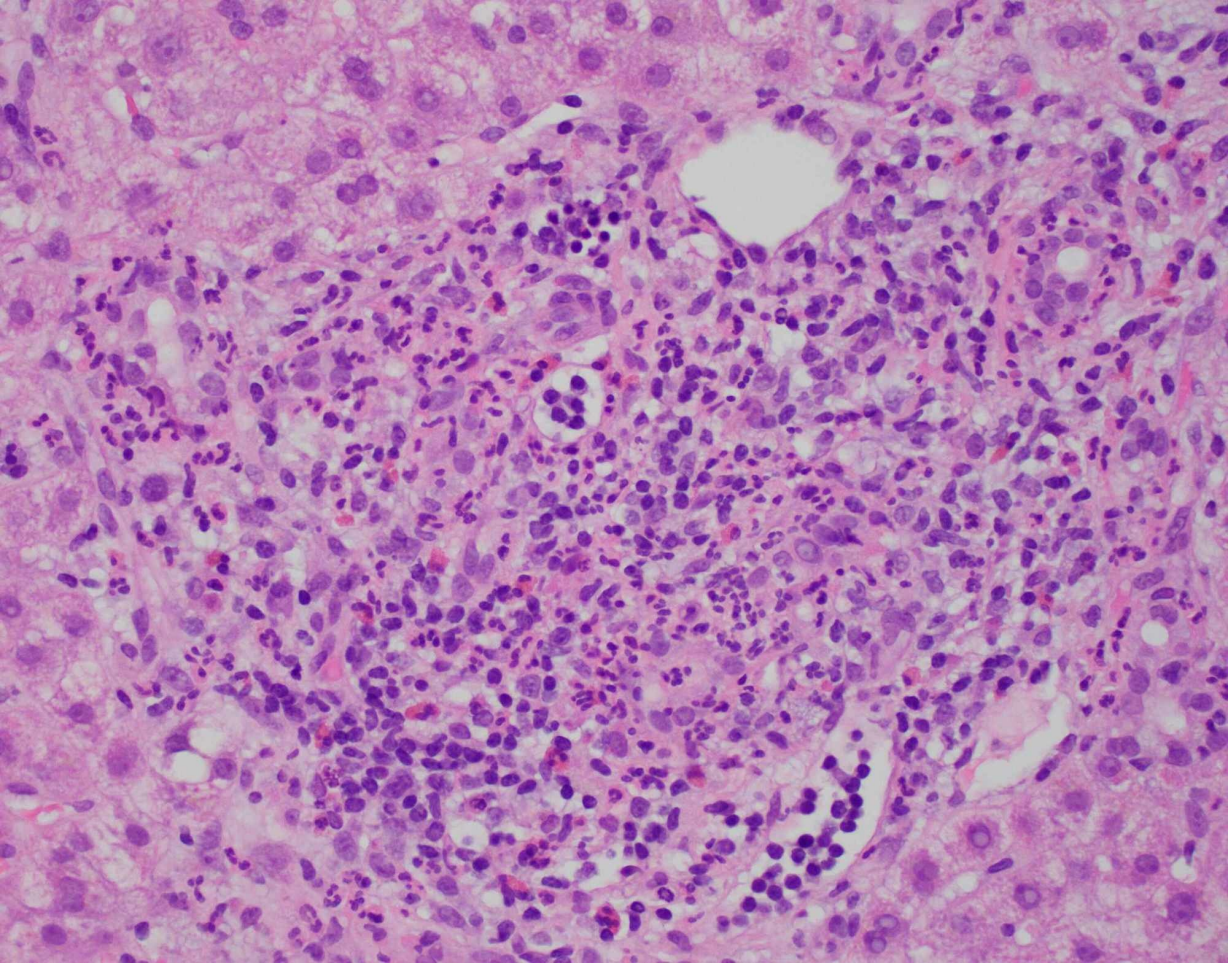
Mixed portal inflammation with eosinophils.

**Figure 6:**
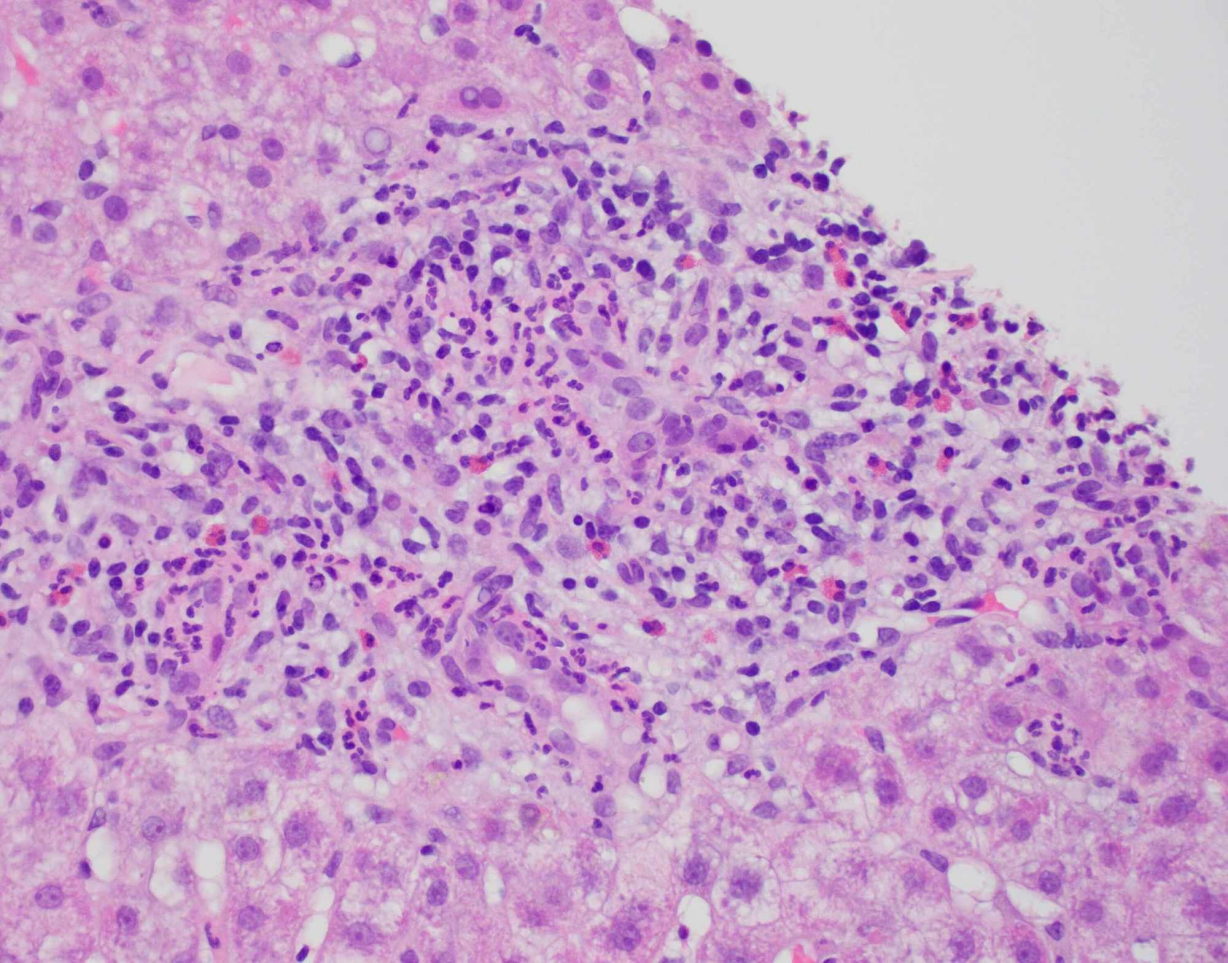
Portal mixed inflammation with eosinophils.

**Figure 7:**
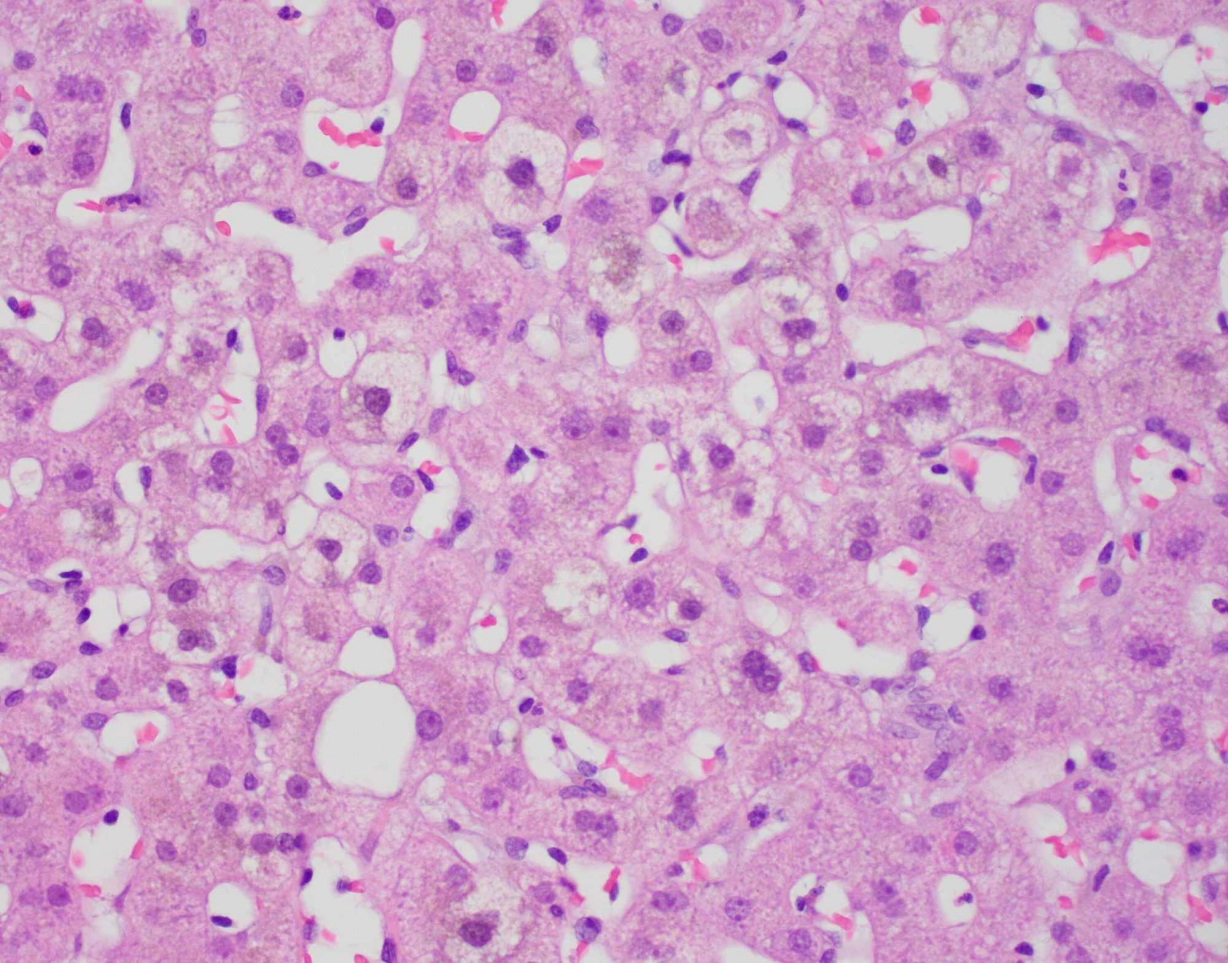
H&E cholestasis. H&E, hemotoxylin and eosin

Diagnostic imaging for the patient included an ultrasound of the abdomen and CT of abdomen and pelvis which showed a contracted and dilated gallbladder to 6 mm (Figure 1). Magnetic resonance cholangiopancreatography (MRCP) was negative for any extra- or intra-hepatic biliary obstruction so a liver biopsy was subsequently obtained (Figure 2). Histopathological examination revealed dense portal infiltrates dominated by neutrophils in the periductal region, but some within bile duct lumens. Substantial numbers of eosinophils accompanied the process in the periductal region. Figures 3-7 showed a prominent acute cholangitis composed of predominantly neutrophils. However, the biopsy was also remarkable for an increased number of accompanying eosinophils. This inflammatory infiltrate occupied Zone 1 and Zone 3 with relative sparing of the hepatic lobules, although there was a small amount of interface hepatitis likely contributing to the patient's elevated liver function tests (LFTs). The hepatic lobules were remarkable for moderate cholestasis. Overall hepatic lobules architecture was preserved, and there was no appreciable background steatosis or fibrosis. There was no evidence of autoimmune hepatitis as pathological and serological examinations were negative. There was no evidence of viral cytopathic effect. Reticulin and trichrome stains were unremarkable. Periodic acid Schiff-hematoxylin (PAS-H) and periodic acid Schiff-diastase stain (PAS-D) did not highlight any discrete intracytoplasmic globules. Iron was inconspicuous with the iron stain.

The histology from the liver biopsy was most consistent with acute large duct obstruction complicated by ascending cholangitis or drug-induced cholestatic hepatitis. However, imaging studies showed no evidence of calcified stones and no ductal dilation, which led to medication, illicit drug, and toxin involvement becoming the leading differential diagnosis. Upon further investigation of the patient's home medication list and a more thorough medication reconciliation a prescription for amoxicillin-clavulanate was found. The patient was treated approximately four months prior to admission for sinusitis with amoxicillin-clavulanate 825 mg/125 mg every 12 hours for 10 days. A corroborating history of present illness, this newly discovered antibiotic subscription, blood test, and biopsy results supported the probable diagnosis of drug-induced cholestatic hepatitis. 

The patient was started on prednisone 40 mg daily and ursodiol 300 mg TID (four months after the last dose of amoxicillin-clavulanate was ingested). Following the commencement of treatment, improvement in clinical status and laboratory studies was seen. The patient was discharged and followed up as an outpatient with serial weekly labs. 

## Discussion

In 1984, amoxicillin-clavulanate was commercialized for the first time. The first-ever reported case of toxic hepatitis related to this antibiotic was from The Netherlands in 1988 [5, 12]. Amoxicillin-clavulanate is generally a well-tolerated antibiotic used for mild to moderate respiratory and cutaneous bacterial infections [12]. Amoxicillin as a single agent is rarely known to cause liver disturbances, however, it was suggested that the addition of clavulanic acid with amoxicillin may possibly be responsible for the adverse reaction [4].

The onset of the symptoms appears soon after completion of the therapy, typically appearing between 4 and 10 weeks after treatment [4, 9-10, 12]. Presenting symptoms include nausea, vomiting, fatigue, generalized malaise, jaundice, pruritus, decreased appetite, dark urine, and light-colored stool suggestive of cholestatic pattern. Other less commonly reported symptoms are the appearance of skin rash and fever. Abdominal examination may show painful hepatomegaly which is also less common.

While the exact pathophysiology of amoxicillin-clavulanate induced hepatotoxicity is still unknown -- eosinophilic infiltrates, skin rash, hypereosinophilia, and positive diagnostic autoantibodies (mitochondrial type 6, anti LKM2, and anti LM antibodies) reinforce the hypothesis of immunoallergic mechanisms caused by the drug [5, 12].

The pattern of amoxicillin-clavulanate associated hepatic damage is predominantly cholestatic, but patients can also present with hepatocellular or mixed cholestatic/hepatocellular patterns. Liver biopsy findings include centrilobular or pan lobular cholestasis with predominant lymphocytic infiltration along with neutrophils and eosinophils. Cholestasis mainly involves portal and periportal areas [4-5]. Ductopenia, necrosis and degeneration of ductal epithelial cells, and vacuolization and necrosis of liver cells are some other findings besides the granulomatous inflammatory process [5, 13-15].

Drug-induced liver injury (DILI) frequently causes diagnostic dilemmas and is often overlooked in medical practice. Diagnosis of DILI includes multifaceted approaches such as establishing a causal relationship between the pharmacotherapy and onset of clinical manifestations, obtaining a detailed pharmacological history, excluding other causes of hepatotoxicity, biochemical profiles, and histological findings.

No targeted treatment for amoxicillin-clavulanate induced hepatic damage is available apart from symptomatic management. Early recognition and prompt cessation of offending agents can prevent further injury [9]. Few studies have indicated that ursodeoxycholic acid (UDCA) arrested drug-induced cholestasis in two-third of cases [16].

Some authors also advocated the use of steroid treatment in patients with high bilirubin levels, LFT abnormalities, and neurological impairment [17]. Their efficacy is yet to be determined in a randomized controlled trial setting [18].

Our patient manifested with nausea, vomiting, epigastric pain, and yellowish discoloration of skinfour months after her last dose of amoxicillin-clavulanate. Her bilirubin, alkaline phosphatase, and transaminase levels continued to rise with no improvement in clinical symptoms. Other causes of liver injury including obstructive jaundice were ruled out. Based on the Council for International Organizations of Medical sciences Score (CIOMS) of +8 points (suggestive of very likely association), The Clinical Diagnostic Scale (CDS) of +11 points (suggestive of possible association), Adverse Drug Reaction Probability (Naranjo) score of +6 points (suggestive of probable association) along with the presence of dense portal infiltrate dominated by neutrophils, eosinophils in the periductal region- our patient had a high probability of DILI. 

The patient did not have any improvement in her clinical symptoms during the observation period. She also developed progressive worsening of cholestasis. The patient was started on a tapering dose of prednisone 40 mg daily and UDCA 300 mg three times a day. The therapy resulted in quick resolution of symptoms as well as jaundice. 

The exact mechanism of steroid therapy remains enigmatic. Nevertheless, the close association between initiation of steroid therapy and improvement in LFTs implies that corticosteroid may be a plausible salutary option for some patients with DILI. As of this publication only a few cases of drug-related cholestatic hepatitis managed successfully with steroids have been reported [5, 17, 19-20]. A pertinent factor which led to a diagnostic dilemma in this case was the patient's denial of using any prescription drug or medication in recent past. This case also emphasized the need for through medication reconciliation upon admission to the acute care setting to avoid diagnostic delays and dilemmas especially in patient with drug induced causes. .

## Conclusions

In conclusion, the amoxicillin-clavulanic acid combination may cause delayed cholestatic hepatitis. Though the mechanism is not clear as of yet, it is suggested to be an immune-allergenic or an idiosyncratic reaction. The symptoms could appear weeks after the discontinuation of the medication, however, it is reassuring that the clinical course is benign with a good prognosis.
